# Mutual Interaction of Basophils and T Cells in Chronic Inflammatory Diseases

**DOI:** 10.3389/fimmu.2015.00399

**Published:** 2015-08-03

**Authors:** Marika Sarfati, Keiko Wakahara, Laurence Chapuy, Guy Delespesse

**Affiliations:** ^1^Centre de Recherche du Centre Hospitalier de l’Université de Montréal (CRCHUM), Université de Montréal, Montréal, QC, Canada; ^2^Department of Respiratory Medicine, Nagoya University Graduate School of Medicine, Nagoya, Japan

**Keywords:** basophils, memory T cells, Th17 cells, Th2 cells, inflammatory bowel diseases

## Abstract

Basophils are, together with mast cells, typical innate effector cells of allergen-induced IgE-dependent allergic diseases. Both cell types express the high-affinity receptor for IgE (FcεR1), release histamine, inflammatory mediators, and cytokines following FcεR1 cross-linking. Basophils are rare granulocytes in blood, lymphoid, and non-lymphoid tissues, and the difficulties to detect and isolate these cells has hampered the study of their biology and the understanding of their possible role in pathology. Furthermore, the existence of other FcεR1-expressing cells, including professional Ag-presenting dendritic cells, generated some controversy regarding the ability of basophils to express MHC Class II molecules, present Ag and drive naïve T cell differentiation into Th2 cells. The focus of this review is to present the recent advances on the interactions between basophils and peripheral blood and tissue memory Th1, Th2, and Th17 cells, as well as their potential role in IgE-independent non-allergic chronic inflammatory disorders, including human inflammatory bowel diseases. Basophils interactions with the innate players of IgE-dependent allergic inflammation, particularly innate lymphoid cells, will also be considered. The previously unrecognized function for basophils in skewing adaptive immune responses opens novel perspectives for the understanding of their contribution to the pathogenesis of inflammatory diseases.

## Basophils in Type 2 Immune Responses

Basophils represent <1% of all blood leukocytes. Together with mast cells, they are regarded as typical effector cells of IgE-dependent allergic inflammation (1, 2). Because both types of cells express high levels of the high-affinity receptor for IgE (FcεR1), and rapidly release histamine and inflammatory mediators upon cross-linking of FcεR1 by IgE-allergen complexes, basophils were long considered as redundant granulocytes lacking unique functions (3). Until recently, the investigations of the functional properties of basophils have been hampered by the difficulty of detecting and purifying these rare cells in blood and tissues from mice and humans (4). Despite the development of transgenic mice (IL-4-eGFP, Basoph8) that allow the tracking or transient depletion of basophils *in vivo*, conflicting results were generated regarding the antigen-presenting function of basophils and their ability to trump DCs in the priming of Th2 responses (5–8). However, it was reported that cooperation between highly purified murine basophils and DCs, isolated from blood or lungs, is required to induce *in vitro* Th2 cell differentiation (9, 10). In this context, DCs ensure naïve T cell proliferation and basophils provide IL-4 to drive Th2 polarization. As a consequence, a careful exclusion of trace amounts of MHC class II^+^FcεR1^+^ DCs from basophils preparation is mandatory to draw valid conclusions about any yet unrecognized *in vitro* function of these rare cells, especially in human studies (11).

Despite their low frequency in blood, increased numbers of basophils were detected in the tissues of several IgE-dependent allergic diseases that include allergic rhinitis, atopic dermatitis, and asthma (2, 12). How basophils are attracted to the site of allergen challenge remains to be clarified. Histamine and PGD2, which are produced by mast cells, as well as IL-3 secreted by activated T cells have been proposed to play crucial roles in the recruitment of basophils to tissues, since basophils express histamine receptors, CRTH2 (i.e., PGD2 receptor) and IL-3 receptors (13–15). In tissues, basophils may interact with resident memory T cells, as demonstrated by two-photon microscopy (8). Hence, prolonged contacts between basophils and T cells occurred in the inflamed lungs but not in the mediastinal lymph nodes (LNs) of parasite-infected mice. The same study also revealed that activated T cells induced IL-4 secretion by basophils in affected lungs. Optimal IL-4 production by basophils required a direct cell/cell contact, as well as the presence of IL-3, a cytokine that promotes expansion and survival of basophils. Conversely, *in vitro* interactions between pulmonary basophils and lung CD4^+^ T cell promoted IL-4-dependent T cell survival and amplified release of Th2 cytokines, without inducing memory T cell expansion (9). In experimental asthma, transfer of lung basophils worsens ongoing Th2 responses by increasing airway inflammation and local IL-4 and IL-13 expression (9). Furthermore, human basophils increase IL-4 expression in effector memory T cells *in vitro*. Notably, responding T cells mainly included CRTH2^+^ cells, corroborating the *in vivo* and *in vitro* data seen in mice. In addition to Th2 cells that produce IL-4, IL-5, and IL-13, double IL-4- and IL-17-expressing memory CD4^+^ T cells were detected in severe asthmatic patients and in lungs of mice developing experimental asthma (16). In contrast, human basophils were not reported to promote the generation of Th2/Th17 double positive cells. The basophil enhancing effect on memory Th2 responses was partially contact dependent, but did not involve OX40/OX40L, CTLA4/B7family, and CD2/LFA3 interactions. Because IL-3-stimulated basophils express RANKL and activated Th cells may express RANK (17, 18), we speculate that RANK–RANKL pairs of molecules may represent other potential candidates involved in basophil-T cell interactions.

Cellular aggregates made of basophils and memory CD4^+^ T cells were detected in the dermis of patients with atopic dermatitis and in a mouse model of TSLP-mediated contact dermatitis, underlying the key role of basophils in T cell-mediated skin allergic disorders (19). However, basophils may regulate type 2 inflammatory responses by interacting with cells other than T cells. Indeed, innate immune cells, such as macrophages, innate lymphoid type 2 (ILC2), and eosinophils, are major contributors of allergic lung and skin inflammatory responses (20, 21). Moreover, keratinocyte-derived TSLP activates basophils that results in local recruitment, activation, and proliferation of ILC2, which is mediated by basophil-derived IL-4 (19). This mechanism initiates experimental atopic dermatitis and appears essential for the development of food allergy induced by the application of food antigen to inflamed skin (22). Furthermore, the interactions of activated basophils with ILC2, fibroblasts, and/or endothelial cells regulate recruitment of eosinophils in experimental models of contact dermatitis and allergic asthma (21, 23).

Taken collectively, these finding indicate that, although basophils do not appear to initiate IgE-dependent allergic disease, the interaction between basophil and memory T cell in inflamed tissues may be bidirectional, thus contributing to the exacerbation of chronic allergic airway inflammation at late stages. In support of this, asthmatic patients are successfully treated by administration of Omalizumab, a monoclonal antibody to IgE that prevents IgE binding to FcεR1 and regulates basophil homeostasis (24). It has been suggested that its therapeutic efficacy largely result from its effect on basophils, since these cells have a much shorter life than mast cells. In fact, the improved clinical outcome of allergic patients following Omalizumab therapy was associated with a reduction in circulating basophil numbers (24).

## Basophils in IgE-Independent Th Responses

Basophils were historically associated exclusively with IgE-dependent allergic disorders. However, TSLP-activated basophils induce and perpetuate experimental eosinophilic esophagitis (EoE), which may be triggered in the absence of IgE and mast cells (25). Notably, the circulating number of basophils is increased in patients with EoE. Similarly, increased numbers of basophils are observed in blood of patients with chronic inflammatory bowel diseases (IBD) (26). Basophilia is found in both Crohn’s disease (CD) and ulcerative colitis (UC), the two main chronic relapsing IBD types that are associated with Th17/Th1 and Th17 or Th2 cells, respectively (27). Strikingly, increased percentages of basophils, but not of mast cells are found in the inflamed relative to the non-inflamed colonic mucosa in both CD and UC patients (26). In contrast, basophils are not detected in the intestinal mucosa of non-IBD individuals. The accumulation of basophils in the inflamed colons of IBD patients suggests that these rare cells contribute to disease pathogenesis by influencing pathogenic T cell responses in tissues.

Indeed, similar to their ability to amplify human memory Th2 responses, basophils promote memory Th17 responses *in vitro* (11). Blood basophils as well as basophils isolated from inflamed colonic mucosa or the mesenteric LNs of IBD patients favor the emergence of memory IL-17^+^, IL-17^+^/IFN-γ^+^ but not IFN-γ^+^ single positive Th cells (26). Activation of CD4^+^ T lymphocytes generates functionally distinct antigen-experienced T cells, namely, effector memory CD62L^low^CCR7^−^ (T_EM_) that migrate to peripheral tissues and central memory CD62L^high^CCR7^+^ (T_CM_) T cells, which retain the ability to enter LNs (28). Thus, basophils activated by either IL-3, IL-33, or TSLP increase Th17 and Th17/Th1 responses by IL-2-stimulated T_EM_ in the absence of APC, as well as by TCR-stimulated T_CM_ isolated from blood, which mimic memory T cell activation in mucosa and lymphoid tissues, respectively (11). More specifically, basophils promote cytokine production by autologous T_EM_ cells in a contact-independent manner that involves the ERK1/2-pathway. Basophil-derived histamine partially increases IL-17 expression through H_2_ and H_4_, but not H_1_ receptors. Interestingly, histamine alone cannot replace basophils in their pro-Th17 activity.

Basophils also enhance IL-22 production by T_EM_ cells but not T_CM_ (11). However, Sharma et al. (29, 30) reported that human basophils lack the ability to drive IL-22 or IL-17 memory CD4^+^ T cell responses. The reasons for the apparent discrepancies might at least result from the analysis of unfractionated stimulated CD45RO^+^CD25^−^ CD4^+^ T cells, which mainly comprises T_CM_, in co-culture with IL-3-activated basophils. Also, the purity of basophils was 94 ± 5% in the latter studies (29, 30), in contrast to >99% purity in the former studies (11, 26), further highlighting the importance of assessing highly purified basophil preparations.

Lymphoid tissues represent the major site of memory T cells in the body relative to circulating pool (31). It is therefore essential, whenever feasible in humans, to assess the functions of T cells that are found in tissues. Basophils enhance Th17 and Th17/Th1 responses by T_CM_ and T_EM_ CD4 T cells isolated from mLNs of IBD patients (26). Furthermore, a small number of tissue memory T_EM_ express CCR7 in inflamed tissues and CCR7^+^ T_EM_ cells are prone to exit the tissues and re-circulate (32, 33). Notably, CCR7^+^ T_EM_ cells are the preferential targets of basophils for enhancing Th17 responses (26). A recent study demonstrates that CCR7 expression controls intestinal Th17 and Th1 balance in a model of TNF-α driven Crohn’s-like ileitis (34). The CCR7-deficient mice or mice treated with anti-CCR7 mAb develop an exacerbated ileo-colitis, which is associated with retention of Th effectors in intestinal and extra-intestinal tissues, suggesting that recirculation of CCR7^+^ T_EM_ contribute to intestinal homeostasis.

## Immunoregulatory Activity of Basophils

Although basophils clearly amplify allergic skin and airway inflammation, several studies showed that these rare cells might also exert anti-inflammatory activities in the context of autoimmune disease, contact dermatitis, as well as colitis. In a mouse model of arthritis, IL-4 production by IL-33-activated basophils was found to enhance expression of the inhibitory Fcγ Receptor (FcγRIIb) on inflammatory macrophages and mediates the immunosuppressive response elicited by injection of intravenous immunoglobulin (IVIG) (35). A recent report indicates that upon IL-33 and IgE triggering, human basophils down-modulate monocyte activation *in vitro* (36). Furthermore, basophil-derived IL-4 significantly attenuates the intensity of skin inflammation by mediating the differentiation of inflammatory monocytes into alternatively activated M2 macrophages that are endowed with an immunosuppressive and anti-inflammatory activity (37).

Basophils display immunoregulatory activity by enhancing the suppressive activity of FOXP3-expressing regulatory T cells (Treg) *in vivo* and *in vitro* (38). In a mouse model of contact hypersensitivity, basophils mediate UVB-induced immune suppression by increasing Treg function and this immunosuppressive effect is reduced in amphiregulin (AREG)-deficient mice (39). Indeed, basophils express AREG, an epidermal cell growth factor-like cytokine that enhances Treg function (40). Finally, the *in vivo* role of basophils in pathologies affecting the gastrointestinal tract remains to be clarified. A direct pathogenic role has been proposed for murine basophils in experimental EoE, as well as in allergen-induced colitis in a humanized mice model (25, 41). On the other hand, basophils depletion aggravates colitis induced by adoptive transfer of T cells in lymphopenic mice (42).

## Concluding Remarks

Overall, the *in vivo* function of mucosal or lymphoid human basophils warrants further investigations in allergic diseases, CD, and UC. Basophils accumulate in inflamed tissues in IgE-dependent, as well as IgE-independent inflammatory disorders whereby they may directly interact with memory T cells to augment Th2, Th17, and Th17/Th1 effector responses. The accumulation of basophils in tissues indicates that they may contribute to the aggravation and flare up of the disease. Conversely, their increased numbers may as well reflect a negative regulatory feedback mechanism to dampen inflammation. Nonetheless, basophils represent an attractive therapeutic target for patients with chronic inflammatory disorders. This opens therapeutic avenues by targeting basophils and histamine using the clinically safe non-degranulating anti-IgE human monoclonal antibody (Omalizumab) (43) or selective anti-histamine receptor drugs (44).

## Conflict of Interest Statement

The authors declare that the research was conducted in the absence of any commercial or financial relationships that could be construed as a potential conflict of interest.
